# Association of α1-Blocker Receipt With 30-Day Mortality and Risk of Intensive Care Unit Admission Among Adults Hospitalized With Influenza or Pneumonia in Denmark

**DOI:** 10.1001/jamanetworkopen.2020.37053

**Published:** 2021-02-10

**Authors:** Reimar W. Thomsen, Christian Fynbo Christiansen, Uffe Heide-Jørgensen, Joshua T. Vogelstein, Bert Vogelstein, Chetan Bettegowda, Suzanne Tamang, Susan Athey, Henrik Toft Sørensen

**Affiliations:** 1 Department of Clinical Epidemiology, Aarhus University Hospital, Aarhus, Denmark; 2 Department of Biomedical Engineering, Institute of Computational Medicine, Johns Hopkins University, Baltimore, Maryland; 3 Department of Biostatistics, Bloomberg School of Public Health, Johns Hopkins University, Baltimore, Maryland; 4 Ludwig Center, Lustgarten Laboratory, Howard Hughes Medical Institute, Johns Hopkins Kimmel Cancer Center, Baltimore, Maryland; 5 Johns Hopkins University School of Medicine, Baltimore, Maryland; 6 Center for Population Health and Sciences, Stanford University, Stanford, California; 7 Stanford Graduate School of Business, Stanford University, Stanford, California

## Abstract

**Importance:**

Alpha 1–adrenergic receptor blocking agents (α1-blockers) have been reported to have protective benefits against hyperinflammation and cytokine storm syndrome, conditions that are associated with mortality in patients with coronavirus disease 2019 and other severe respiratory tract infections. However, studies of the association of α1-blockers with outcomes among human participants with respiratory tract infections are scarce.

**Objective:**

To examine the association between the receipt of α1-blockers and outcomes among adult patients hospitalized with influenza or pneumonia.

**Design, Setting, and Participants:**

This population-based cohort study used data from Danish national registries to identify individuals 40 years and older who were hospitalized with influenza or pneumonia between January 1, 2005, and November 30, 2018, with follow-up through December 31, 2018. In the main analyses, patients currently receiving α1-blockers were compared with those not receiving α1-blockers (defined as patients with no prescription for an α1-blocker filled within 365 days before the index date) and those currently receiving 5α-reductase inhibitors. Propensity scores were used to address confounding factors and to compute weighted risks, absolute risk differences, and risk ratios. Data were analyzed from April 21 to December 21, 2020.

**Exposures:**

Current receipt of α1-blockers compared with nonreceipt of α1-blockers and with current receipt of 5α-reductase inhibitors.

**Main Outcomes and Measures:**

Death within 30 days of hospital admission and risk of intensive care unit (ICU) admission.

**Results:**

A total of 528 467 adult patients (median age, 75.0 years; interquartile range, 64.4-83.6 years; 273 005 men [51.7%]) were hospitalized with influenza or pneumonia in Denmark between 2005 and 2018. Of those, 21 772 patients (4.1%) were currently receiving α1-blockers compared with a population of 22 117 patients not receiving α1-blockers who were weighted to the propensity score distribution of those receiving α1-blockers. In the propensity score–weighted analyses, patients receiving α1-blockers had lower 30-day mortality (15.9%) compared with patients not receiving α1-blockers (18.5%), with a corresponding risk difference of −2.7% (95% CI, −3.2% to −2.2%) and a risk ratio (RR) of 0.85 (95% CI, 0.83-0.88). The risk of ICU admission was 7.3% among patients receiving α1-blockers and 7.7% among those not receiving α1-blockers (risk difference, −0.4% [95% CI, −0.8% to 0%]; RR, 0.95 [95% CI, 0.90-1.00]). A comparison between 18 280 male patients currently receiving α1-blockers and 18 228 propensity score–weighted male patients currently receiving 5α-reductase inhibitors indicated that those receiving α1-blockers had lower 30-day mortality (risk difference, −2.0% [95% CI, −3.4% to −0.6%]; RR, 0.89 [95% CI, 0.82-0.96]) and a similar risk of ICU admission (risk difference, −0.3% [95% CI, −1.4% to 0.7%]; RR, 0.96 [95% CI, 0.83-1.10]).

**Conclusions and Relevance:**

This cohort study’s findings suggest that the receipt of α1-blockers is associated with protective benefits among adult patients hospitalized with influenza or pneumonia.

## Key Points

**Question:**

Is the receipt of α1–adrenergic receptor blocking agents (α1-blockers) associated with protective benefits against adverse outcomes, such as mortality and intensive care unit admission, among adult patients with severe respiratory tract infections?

**Findings:**

In this cohort study of 528 467 Danish adults hospitalized with influenza or pneumonia, current receipt of α1-blockers was associated with a 14% reduction in the relative risk of 30-day mortality.

**Meaning:**

This study’s findings suggest that the receipt of α1-blockers may have a clinically relevant association with protective benefits against adverse outcomes among patients with severe respiratory tract infections.

## Teaser

This cohort study uses data from national registries to examine the association between the receipt of α1–adrenergic receptor blockers and the risks of 30-day mortality and intensive care unit admission among adult patients hospitalized with influenza or pneumonia in Denmark between 2005 and 2018.

## Introduction

Acute respiratory syndrome coronaviruses are associated with severe viral pneumonia and death.^1^ In the ongoing coronavirus disease 2019 (COVID-19) pandemic, mortality appears to be associated with acute respiratory distress syndrome and a dysregulated immune response with hyperinflammation and cytokine storm syndrome,^2,3^ factors that have also been observed in patients with other severe respiratory tract infections and sepsis.^4,5^ In mouse models, α1–adrenergic receptor blocking agents (α1-blockers), which are mainly used to treat benign prostatic hyperplasia (BPH) and hypertension, have recently been reported to protect against hyperinflammation and cytokine storm syndrome after exposure to various inflammatory stimuli.^6,7,8^

Given the safety profile and low cost of treatment with α1-blockers, any benefits associated with protection against adverse outcomes among patients hospitalized with COVID-19 or other severe respiratory tract infections would have substantial clinical and public health importance.^9^ Studies of the association of α1-blockers with outcomes among human study participants with respiratory tract infections are scarce or nonexistent.^8^ To address this gap, we conducted a large population-based study using data from Danish national registries to investigate the association of the receipt of α1-blockers with intensive care unit (ICU) admission and 30-day mortality among patients hospitalized with influenza or pneumonia.

## Methods

### Study Design and Setting

The population for this nationwide cohort study included all patients 40 years and older who were hospitalized with influenza or pneumonia in Denmark between January 1, 2005, and November 30, 2018, with follow-up through December 31, 2018 (eFigure 1 in the Supplement). Data collection and processing were reported to the Danish Data Protection Agency through Aarhus University. Ethics review board approval and informed consent are not required for registry-based observational studies in Denmark. This study followed the Strengthening the Reporting of Observational Studies in Epidemiology (STROBE) reporting guideline for cohort studies.^10^

Denmark has a tax-supported health care system that provides health care services, including acute care and hospital care for influenza and pneumonia, to all residents.^11^ All Danish residents receive a personal identity number at birth or immigration that allows individual-level linkage across the extensive Danish registry system, which includes national-level information on residence; prescriptions; vital status (dead or alive); and primary, specialty, and hospital-based care.^12^

**Figure 1.**
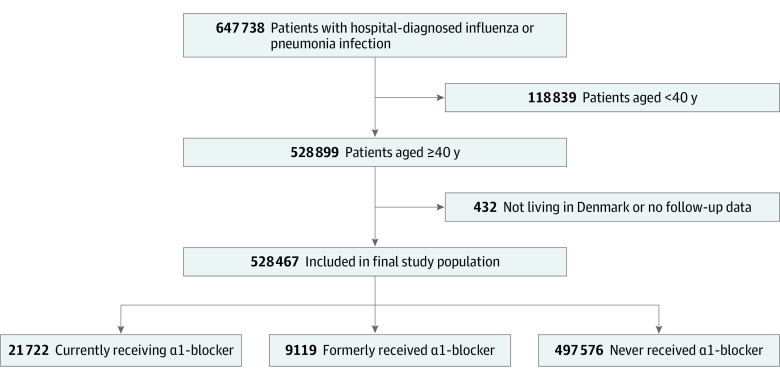
Patient Flow Diagram α1-blocker indicates α1–adrenergic receptor blocking agent.

We assessed hospitalizations (including direct inpatient hospital admissions and emergency department visits leading to either inpatient hospital admission or discharge to home) among patients 40 years and older (because receipt of α1-blockers is rare among individuals younger than 40 years) who had either a primary or secondary diagnosis of influenza or pneumonia recorded in the Danish National Patient Registry (Figure 1).^13^ This registry includes data on primary and secondary diagnoses; procedure codes; and dates of hospital contacts; admissions, and discharges. Hospitalizations that were preceded by an influenza or pneumonia diagnosis within the previous 3 months were excluded to avoid the inclusion of readmissions. We predefined subgroups based on an influenza diagnosis or a diagnosis of pneumonia that specified a bacterial or nonspecific pathogen. Specific diagnostic codes used in the study are listed in eTable 1 in the Supplement.

### Outcomes and Exposures

Primary study outcomes were 30-day mortality and 30-day ICU admission during the index hospitalization associated with an influenza or pneumonia diagnosis. Secondary outcomes included the receipt of organ-supportive treatment (mechanical ventilation, noninvasive ventilation, and treatment with inotropic and/or vasopressor medications) during ICU admission. Dialysis-treated acute kidney injury was defined as treatment with acute renal replacement therapy among patients with no history of previous dialysis for the treatment of chronic kidney disease. Outcomes were ascertained using population registry^1^ data for all-cause death and patient registry^2^ data for diagnoses and procedures associated with all other outcomes.^12,13,14^

Data on all filled prescriptions were obtained from the Danish National Prescription Registry.^15^ This registry contains data on all prescription drugs obtained by Danish residents at any community pharmacy in Denmark since 1995. The main exposure of interest was current receipt of α1-blockers, which are primarily used for 2 indications: BPH and hypertension (eTable 1 in the Supplement). We defined current receipt of α1-blockers as a prescription filled within 90 days before a hospitalization for influenza or pneumonia. This definition was consistent with the 3-month supply of α1-blockers that is dispensed most often in Danish pharmacies.

In our main analyses, patients currently receiving α1-blockers were compared with those not receiving α1-blockers (defined as patients with no prescription for an α1-blocker filled within 365 days before the index date). In a secondary analysis, patients who formerly received α1-blockers (defined as patients with a prescription for an α1-blocker that was filled 91-365 days before the index date) were compared with those who were not receiving α1-blockers to address potential confounding by treatment indication. In an additional analysis to account for confounding by treatment indication, patients currently receiving α1-blockers were compared with those currently receiving a different type of medication, 5α-reductase inhibitors (finasteride and dutasteride), for the treatment of BPH. This analysis was restricted to male patients who did not receive the 2 types of drugs (α1-blockers and 5α-reductase inhibitors) as combination therapy for BPH.

### Potential Confounders

We considered a range of potential confounders in our study.^16^ We obtained patient age and sex from the population registry,^12^ and we collected information from the patient registry on the presence of a range of comorbidities that required inpatient or outpatient hospital contact within 10 years before the index hospitalization (Table 1; eTable 1 in the Supplement).^13^

**Table 1.** Characteristics of Patients Currently Receiving α1-Blockers vs Patients Not Receiving α1-Blockers^a^

Characteristic	Overall cohort (N = 519 348)			Propensity score–weighted cohort (n = 43 889)		
	Currently receiving α1-blocker, No. (%)		Standardized difference	Currently receiving α1-blocker, No. (%)		Standardized difference
	Yes	No		Yes	No^b^	
Total participants	21 772 (4.2)	497 576 (95.8)	NA	21 772 (49.6)	22 117 (50.4)	NA
Age, median (IQR), y	79.7 (72.8-85.4)	74.6 (63.8-83.5)	0.68	79.7 (72.8-85.4)	79.9 (73.0-85.5)	0.03
Men	20 984 (96.4)	243 314 (48.9)	1.78	20 984 (96.4)	21 329 (96.4)	0
Comorbidities (within previous 10 y)						
Hospital-diagnosed hypertension	9477 (43.5)	156 419 (31.4)	0.36	9477 (43.5)	9783 (44.2)	0.02
Hospital-diagnosed BPH	5960 (27.4)	26 240 (5.3)	0.89	5960 (27.4)	6342 (28.7)	0.04
Previous myocardial infarction	2125 (9.8)	36 951 (7.4)	0.12	2125 (9.8)	2202 (10.0)	0.01
Stable angina pectoris	4277 (19.6)	62 834 (12.6)	0.27	4277 (19.6)	4412 (19.9)	0.01
Heart failure	3884 (17.8)	65 894 (13.2)	0.18	3884 (17.8)	4004 (18.1)	0.01
Stroke	3404 (15.6)	60 921 (12.2)	0.14	3404 (15.6)	3473 (15.7)	0
Atrial fibrillation or flutter	5689 (26.1)	87 846 (17.7)	0.29	5689 (26.1)	5858 (26.5)	0.01
Heart valve disease	2032 (9.3)	31 982 (6.4)	0.15	2032 (9.3)	2098 (9.5)	0.01
Venous thromboembolism	1190 (5.5)	26 370 (5.3)	0.01	1190 (5.5)	1189 (5.4)	0.01
Diabetes	5392 (24.8)	87 330 (17.6)	0.25	5392 (24.8)	5530 (25.0)	0.01
Chronic pulmonary disease	7272 (33.4)	144 482 (29.0)	0.13	7272 (33.4)	7418 (33.5)	0
Kidney disease	3237 (14.9)	35 454 (7.1)	0.35	3237 (14.9)	3415 (15.4)	0.02
End-stage kidney disease	687 (3.2)	7316 (1.5)	0.16	687 (3.2)	747 (3.4)	0.02
Liver disease	385 (1.8)	13 916 (2.8)	0.10	385 (1.8)	392 (1.8)	0
Dementia	1551 (7.1)	33 246 (6.7)	0.02	1551 (7.1)	1605 (7.3)	0.01
Cancer	5312 (24.4)	98 742 (19.8)	0.16	5312 (24.4)	5426 (24.5)	0
Metastatic cancer	762 (3.5)	17 546 (3.5)	0	762 (3.5)	780 (3.5)	0
Peptic ulcer disease	1507 (6.9)	30 312 (6.1)	0.05	1507 (6.9)	1552 (7.0)	0.01
Rheumatoid arthritis or connective tissue disease	1285 (5.9)	30 309 (6.1)	0.01	1285 (5.9)	1299 (5.9)	0
Comedication						
BPH drugs other than α1-blockers	2798 (12.9)	4382 (0.9)	0.69	2798 (12.9)	3087 (14.0)	0.05
5α-Reductase inhibitors	2704 (12.4)	4382 (0.9)	0.67	2704 (12.4)	3087 (14.0)	0.06
Total No. of antihypertensive medications^c^						
0	8580 (39.4)	270 031 (54.3)	0.43	8580 (39.4)	8581 (38.8)	0.02
1	6487 (29.8)	121 347 (24.4)	0.17	6487 (29.8)	6615 (29.9)	0
2	4486 (20.6)	75 009 (15.1)	0.20	4486 (20.6)	4597 (20.8)	0.01
3-6	2219 (10.2)	31 189 (6.3)	0.20	2219 (10.2)	2323 (10.5)	0.01
Antihypertensive medications						
Angiotensin-converting enzyme inhibitor	4507 (20.7)	76 215 (15.3)	0.20	4507 (20.7)	4590 (20.8)	0
Angiotensin II receptor blocker	3014 (13.8)	49 402 (9.9)	0.17	3014 (13.8)	3116 (14.1)	0.01
Calcium channel blocker	4371 (20.1)	65 473 (13.2)	0.26	4371 (20.1)	4521 (20.4)	0.01
Thiazide	3979 (18.3)	74 754 (15.0)	0.12	3979 (18.3)	4075 (18.4)	0.01
β-blocker	6492 (29.8)	102 605 (20.6)	0.30	6492 (29.8)	6726 (30.4)	0.02
Other	312 (1.4)	2545 (0.5)	0.13	312 (1.4)	356 (1.6)	0.02
Other medications						
Statin	6822 (31.3)	104 528 (21.0)	0.33	6822 (31.3)	7015 (31.7)	0.01
Aspirin	7073 (32.5)	111 140 (22.3)	0.32	7073 (32.5)	7277 (32.9)	0.01
Loop diuretic	7538 (34.6)	118 023 (23.7)	0.34	7538 (34.6)	7794 (35.2)	0.02
Antibiotic	5606 (25.7)	134 203 (27.0)	0.04	5606 (25.7)	5732 (25.9)	0.01
Antiviral	42 (0.2)	1162 (0.2)	0.01	42 (0.2)	41 (0.2)	0
Immunosuppressant	280 (1.3)	6057 (1.2)	0.01	280 (1.3)	275 (1.2)	0.01
Glucocorticoid	3822 (17.6)	72 324 (14.5)	0.12	3822 (17.6)	3901 (17.6)	0
Nonsteroidal anti-inflammatory	2694 (12.4)	60 577 (12.2)	0.01	2694 (12.4)	2747 (12.4)	0
Opioid	6201 (28.5)	133 795 (26.9)	0.05	6201 (28.5)	6339 (28.7)	0.01
Vitamin K antagonist	2560 (11.8)	34 655 (7.0)	0.23	2560 (11.8)	2646 (12.0)	0.01
Proton pump inhibitor	7372 (33.9)	134 774 (27.1)	0.21	7372 (33.9)	7613 (34.4)	0.02
Antidepressant	5236 (24.0)	111 203 (22.3)	0.06	5236 (24.0)	5408 (24.5)	0.01
Hypnotic or sedative	3528 (16.2)	73 162 (14.7)	0.06	3528 (16.2)	3647 (16.5)	0.01
Antipsychotic	1434 (6.6)	35 909 (7.2)	0.04	1434 (6.6)	1493 (6.8)	0.01
Lifestyle and social factors						
Smoking	11 629 (53.4)	248 966 (50.0)	0.10	11 629 (53.4)	11 851 (53.6)	0
Obesity	1454 (6.7)	37 759 (7.6)	0.05	1454 (6.7)	1486 (6.7)	0
Alcohol use	1454 (6.7)	42 514 (8.5)	0.10	1454 (6.7)	1442 (6.5)	0.01
Marital status						
Widowed	5317 (24.4)	147 139 (29.6)	0.16	5317 (24.4)	5442 (24.6)	0.01
Divorced	2405 (11.0)	80 651 (16.2)	0.21	2405 (11.0)	2399 (10.8)	0.01
Married	12 487 (57.4)	216 421 (43.5)	0.40	12 487 (57.4)	12 710 (57.5)	0
Unmarried	1563 (7.2)	53 365 (10.7)	0.18	1563 (7.2)	1566 (7.1)	0.01
Urban residence	6540 (30.0)	177 912 (35.8)	0.17	6540 (30.0)	6649 (30.1)	0

Abbreviations: α1-blocker, α1–adrenergic receptor blocking agent; BPH, benign prostatic hyperplasia; IQR, interquartile range; NA, not applicable.

^a^ A total of 9119 patients (1.7%) who formerly received α1-blockers were not included in the table.

^b^ A population of patients not receiving α1-blockers was weighted to the propensity score distribution of patients currently receiving α1-blockers.

^c^ Includes antihypertensive medications with the exception of α1-blockers.

Prescriptions for relevant concurrent medications that were filled within 90 days before hospital admission were also ascertained; these medications included angiotensin-converting enzyme inhibitors, angiotensin II receptor blockers, calcium channel blockers, thiazides, β-blockers, other antihypertensive medications, statins, aspirin, loop diuretics, antibiotics, antiviral agents, glucocorticoids, other immunosuppressants, nonsteroidal anti-inflammatory drugs, opioids, vitamin K antagonists, proton pump inhibitors, antidepressants, hypnotics or sedatives, and antipsychotics.^15^ Because lifestyle and social factors are associated with health, we included information on obesity, alcohol use, smoking, marital status, and urban vs rural residence.

### Statistical Analysis

We applied propensity score balancing of potential confounders across treatment groups.^17^ Continuous covariates were included as a cubic spline with 7 knots. We used propensity score weighting to generate a population of relevant comparison groups (eg, patients not receiving α1-blockers or 5α-reductase inhibitors) that resembled the number and covariate distribution of patients receiving α1-blockers. The exposed patients were assigned a weight of 1, and the unexposed patients were assigned a weight equivalent to their estimated propensity score divided by the difference between 1 and their estimated propensity score (ie, the weight was the individual’s estimated odds of being exposed). If successful, this weighting method produces a comparison population with size and covariate distribution resembling that of the exposed population.^17^ Covariate balance was assessed using standardized differences and was deemed acceptable.

Follow-up started on the date of the first hospital admission associated with an influenza or pneumonia diagnosis and continued until a specific outcome of interest, emigration, or the completion of 30 days, whichever occurred first. The 30-day risks (both unadjusted and weighted by propensity score) of death and ICU admission were computed and plotted. Risk differences were calculated for all outcomes by subtracting propensity score–weighted risks. Risk ratios (RRs) were estimated as the ratios of propensity score–weighted risk estimates. All estimates were accompanied by 95% CIs that were obtained using bootstrapping with 200 bootstrap samples.

Several subgroup analyses were conducted that were stratified by (1) patient age, (2) restriction to patients with a diagnosis of influenza or pneumonia that was listed first in the hospital discharge summary, (3) restriction to patients with BPH or hypertension as underlying conditions, and (4) the 3 most frequently prescribed α1-blocker medications (doxazosin, alfuzosin, and tamsulosin). We also performed an analysis of 30-day risk of death associated with the receipt of α1-blockers that was restricted to patients transferred to the ICU during their hospitalization, with follow-up beginning on the date of ICU admission. In additional analyses, current receipt and nonreceipt of α1-blockers were compared among male patients only.

Statistical analyses were performed using SAS software, version 9.4 (SAS Institute). Data were analyzed from April 21 to December 21, 2020.

## Results

**Figure 2.**
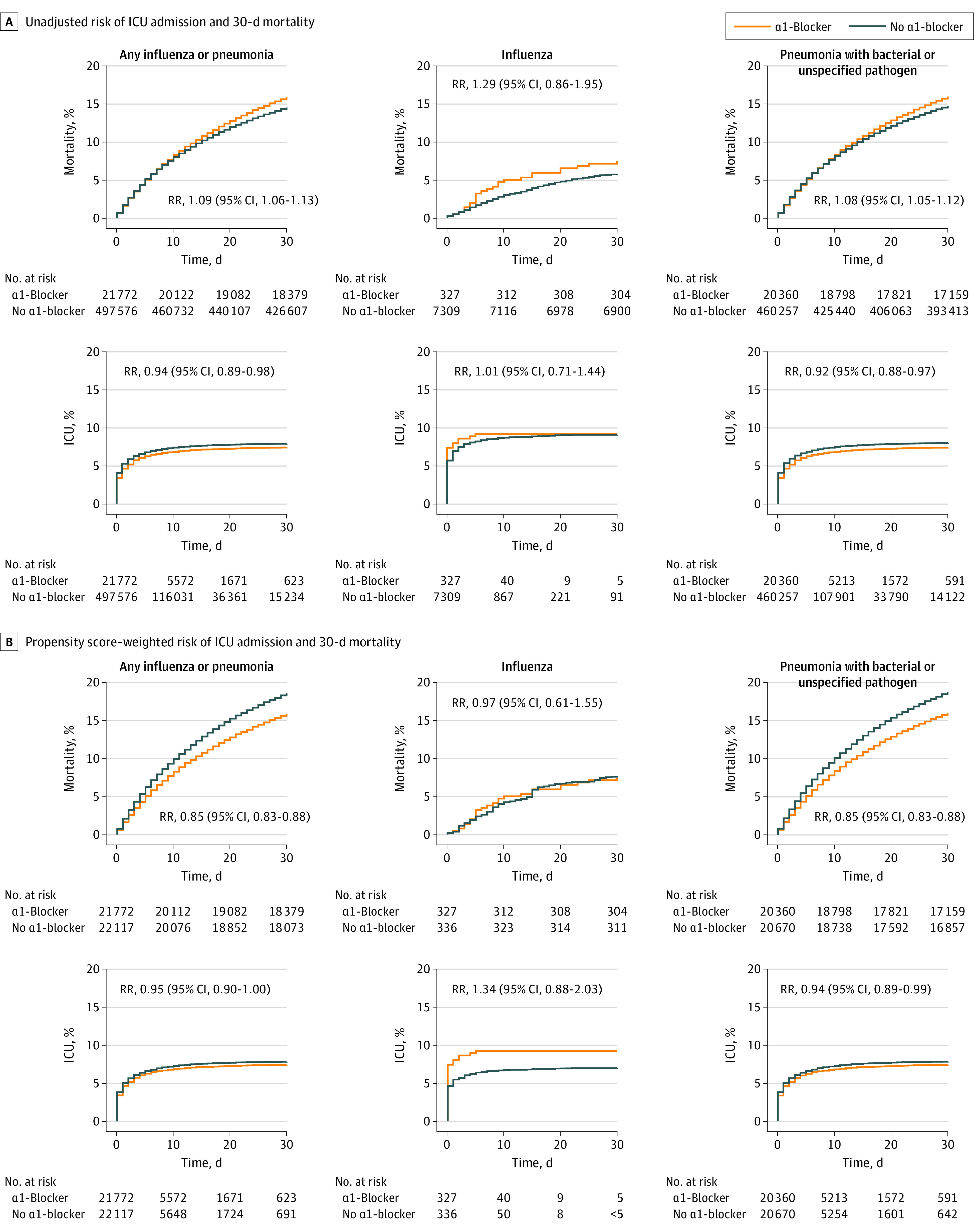
Cumulative Risk of 30-Day Mortality and Intensive Care Unit (ICU) Admission α1-Blocker indicates α1–adrenergic receptor blocking agent and RR, risk ratio.

The final study cohort included 528 467 Danish residents 40 years and older who were hospitalized with influenza or pneumonia (median age, 75.0 years; interquartile range [IQR], 64.4-83.6 years; 273 005 men [51.7%]) (Figure 2; eTable 2 in the Supplement). Of those, 21 772 patients (4.1%) were currently receiving α1-blockers, 9119 patients (1.7%) had formerly received α1-blockers, and 497 576 patients (95.8%) had not received α1-blockers. A total of 41 276 hospitalizations included admission to the ICU; in most cases, transfer to the ICU occurred early after the initial hospital admission (median, 1 day; 25th-75th percentile, 0-5 days). In total, 77 197 patients (14.6%) died within 30 days.

### Patient Characteristics

The median age was higher among patients receiving α1-blockers (79.7 years; IQR, 72.8-85.4 years) compared with patients not receiving α1-blockers (74.6 years; IQR, 63.8-83.5 years). The cohort receiving α1-blockers comprised a substantially larger proportion of male patients (20 984 men [96.4%]) than the cohort not receiving α1-blockers (243 314 men [48.9%]). Higher prevalence of previous hospital-diagnosed BPH (5960 patients [27.4%] vs 26 240 patients [5.3%]), hypertension (9477 patients [43.5%] vs 156 419 patients [31.4%]), and other comorbidities (eg, atrial fibrillation, 5689 patients [26.1%] vs 87 846 patients [17.7%]) were observed among those receiving α1-blockers compared with those not receiving α1-blockers, respectively (Table 1). Cotreatment with most cardiovascular medications, including other antihypertensive drugs, was also more frequent among patients receiving α1-blockers (eg, 2704 patients [12.4%] receiving α1-blockers were also receiving 5α-reductase inhibitors compared with 4382 patients [0.9%] not receiving α1-blockers).

After propensity score weighting of patients receiving α1-blockers (eFigure 2 in the Supplement), treatment groups were well balanced on all measured covariates, with absolute standardized differences for all covariates decreasing from between 0 and 1.78 before propensity score balancing to less than 0.10 after propensity score balancing (Table 1). The final cohorts included in the propensity score–weighted outcome analysis consisted of 21 772 patients currently receiving α1-blockers and 22 117 patients not receiving α1-blockers (weighted to the propensity score distribution of the patients currently receiving α1-blockers).

### Patient Outcomes

**Figure 3.**
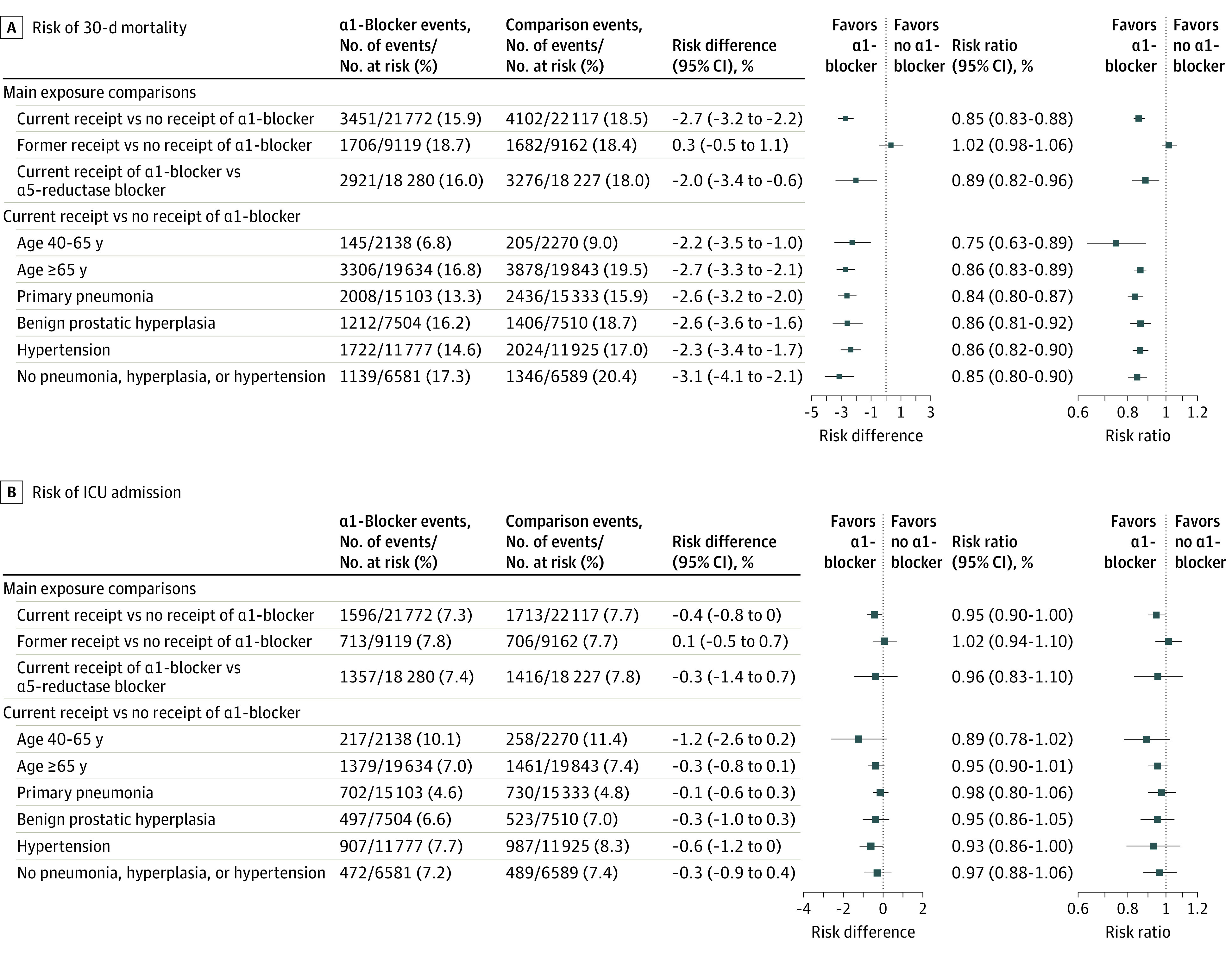
Forest Plot of Risk of 30-Day Mortality and Intensive Care Unit (ICU) Admission

In the unadjusted analyses before propensity score weighting, patients receiving α-1 blockers had higher 30-day mortality (15.9%) than those not receiving α-1 blockers (14.5%) (eTable 3 in the Supplement), which was likely associated with the older age and greater comorbidity burden of this cohort compared with the cohort not receiving α-1 blockers. After covariate balancing by propensity score weighting, among all patients with influenza or pneumonia, 30-day mortality was 15.9% for patients receiving α1-blockers and 18.5% for patients not receiving α1-blockers, with a corresponding risk difference of −2.7% (95% CI, −3.2% to −2.2%) and an RR of 0.85 (95% CI, 0.83-0.88) (Table 2). The risk of ICU admission was 7.3% in patients receiving α1-blockers and 7.7% in those not receiving α1-blockers, which corresponded to a risk difference of −0.4% (95% CI, −0.8% to 0%) and an RR of 0.95 (95% CI, 0.90-1.00). The RRs among patients receiving α1-blockers were almost identical when the analysis was restricted to the risk of ICU admission within 7 days vs 30 days (6.4% vs 6.9%, respectively; risk difference, −0.4% [95% CI, −0.7% to 0%]; RR, 0.94 [95% CI, 0.89-1.00]) (Figure 3). The RR for 30-day ICU admission among those receiving α1-blockers was 0.95 (95% CI, 0.90-1.00), with RRs lower than 1.00 for mechanical ventilation (0.92; 95% CI, 0.86-0.99) and inotropic treatment (0.95; 95% CI, 0.89-1.02) during ICU admission. The RRs for noninvasive ventilation and dialysis-treated acute kidney injury during ICU admission were 1.00 (95% CI, 0.92-1.08) and 1.11 (95% CI, 0.96-1.29), respectively (Table 2).

**Table 2.** Risk of Different Outcomes After Propensity Score Weighting Among Patients Currently Receiving α1-Blockers vs Patients Not Receiving α1-Blockers^a^

Diagnosis	Outcome	Patients currently receiving α1-blockers		Patients not receiving α1-blockers		Risk difference (95% CI), %	Risk ratio (95% CI)
		No. of events/No. at risk	Risk, %	No. of events/No. at risk	Risk, %		
Any influenza or pneumonia	Death	3451/21 772	15.9	4102/22 117	18.5	−2.7 (−3.2 to −2.2)	0.85 (0.83 to 0.88)
	ICU admission	1596/21 772	7.3	1713/22 117	7.7	−0.4 (−0.8 to 0)	0.95 (0.90 to 1.00)
	ICU admission and MV	804/21 772	3.7	883/22 117	4.0	−0.3 (−0.6 to 0)	0.92 (0.86 to 0.99)
	ICU admission and NIV	666/21 772	3.1	678/22 117	3.1	0 (−0.3 to 0.2)	1.00 (0.92 to 1.08)
	ICU admission and inotropic medication	715/21 772	3.3	762/22 117	3.4	−0.2 (−0.4 to 0.1)	0.95 (0.89 to 1.02)
	D-AKI	214/21 085	1.0	195/21 382	0.9	0.1 (0 to 0.2)	1.11 (0.96 to 1.29)
Influenza	Death	24/327	7.3	25/336	7.6	−0.2 (−3.7 to 3.2)	0.97 (0.61 to 1.55)
	ICU admission	30/327	9.2	23/336	6.9	2.3 (−1.3 to 5.9)	1.34 (0.88 to 2.03)
	ICU admission and MV	12/327	3.7	14/336	4.1	−0.4 (−2.6 to 1.7)	0.90 (0.49 to 1.64)
	ICU admission and NIV	18/327	5.5	11/336	3.2	2.3 (−0.5 to 5.1)	1.71 (0.95 to 3.07)
	ICU admission and inotropic medication	12/327	3.7	14/336	4.3	−0.6 (−2.9 to 1.8)	0.86 (0.46 to 1.61)
	D-AKI	NR^b^	NR^b^	NR^b^	NR^b^	−0.3 (−1.1 to 0.4)	0.50 (0.19 to 1.33)
Pneumonia of bacterial or unspecified origin	Death	3256/20 360	16.0	3867/20 670	18.7	−2.7 (−3.3 to −2.2)	0.85 (0.83 to 0.88)
	ICU admission	1483/20 360	7.3	1597/20 670	7.7	−0.4 (−0.8 to 0)	0.94 (0.89 to 0.99)
	ICU admission and MV	742/20 360	3.6	817/20 670	4.0	−0.3 (−0.6 to 0)	0.92 (0.86 to 0.99)
	ICU admission and NIV	613/20 360	3.0	636/20 670	3.1	−0.1 (−0.3 to 0.2)	0.98 (0.90 to 1.06)
	ICU admission and inotropic medication	663/20 360	3.3	707/20 670	3.4	−0.2 (−0.4 to 0.1)	0.95 (0.88 to 1.03)
	D-AKI	196/19 736	1.0	178/20 004	0.9	0.1 (0 to 0.2)	1.11 (0.96 to 1.29)

Abbreviations: α1-blocker, α1–adrenergic receptor blocking agent; D-AKI, dialysis-treated acute kidney injury; ICU, intensive care unit; MV, mechanical ventilation; NIV, noninvasive ventilation; NR, not reported.

^a^ Patients who formerly received α1-blockers were not included in the analysis.

^b^ To ensure anonymity, Danish legislation prohibits the reporting of exact values for instances in which low numbers of participants (eg, n <5) are observed or can be inferred using information from other categories.

In the outcome analysis of the subgroup of 7636 patients diagnosed with influenza (which included 327 patients receiving α1-blockers and 336 propensity score–weighted patients not receiving α1-blockers), 30-day mortality was 7.3% among those receiving α1-blockers and 7.6% among those not receiving α1-blockers (risk difference, −0.2% [95% CI, −3.7% to 3.2%]; RR, 0.97 [95% CI, 0.61-1.55]) (Table 2). The risk of ICU admission was slightly higher among patients with influenza who were receiving vs not receiving α1-blockers (RR, 1.34; 95% CI, 0.88-2.02), whereas the risk of dialysis-treated acute kidney injury was lower (RR, 0.50; 95% CI, 0.19-1.36); however, these estimates were imprecise owing to the limited number of outcomes available for analysis. Outcomes for those receiving vs not receiving α1-blockers were only similar when restricting the analysis to male patients (eTable 4 in the Supplement).

The characteristics of patients currently receiving α1-blockers and those currently receiving 5α-reductase inhibitors were well balanced after propensity score weighting (eTable 5 in the Supplement). In a comparison between 18 280 men receiving α1-blockers and 18 228 propensity score–weighted men receiving 5α-reductase inhibitors, those receiving α1-blockers had lower 30-day mortality (16.0% vs 18.0%, respectively) (Figure 3; eTable 6 in the Supplement). The corresponding risk difference was −2.0% (95% CI, −3.4 to −0.6), and the RR was 0.89 (95% CI, 0.82-0.96). The risk of ICU admission was similar in the 2 groups (7.4% for those receiving α1-blockers and 7.8% for those receiving 5α-reductase inhibitors; risk difference, −0.3% [95% CI, −1.4% to 0.7%]; RR, 0.96 [95% CI, 0.83-1.10], respectively) (eTable 6 in the Supplement; Figure 3). Other outcomes and infection subgroups could not be examined because of sample size constraints.

The subgroup analyses (stratified by age, patients with a first-listed diagnosis of influenza or pneumonia, and patients with BPH or hypertension) were generally consistent with our main findings (Figure 3). An analysis of α1-blockers by type indicated that tamsulosin, alfuzosin, and doxazosin were all associated with reductions in the risk of mortality (for tamsulosin, RR, 0.84 [95% CI, 0.80-0.88]; for alfuzosin, RR, 0.87 [95% CI, 0.82-0.92]; and for doxazosin, RR, 0.92 [95% CI, 0.98-1.00]) and the risk of ICU admission (for tamsulosin, RR, 0.92 [95% CI, 0.86-0.99]; for alfuzosin, RR, 0.94 [95% CI, 0.85-1.04; and for doxazosin, RR, 1.00 [95% CI, 0.90-1.12]) (eTable 7 in the Supplement).

## Discussion

In this large nationwide population-based cohort study of 528 467 Danish patients 40 years or older who were hospitalized with influenza or pneumonia, receipt of α1-blockers was associated with a decreased risk of death, compared with nonreceipt of α1-blockers and receipt of 5α-reductase inhibitors.

This study provides novel information about the association of α1-blockers with protective benefits against adverse outcomes among patients with severe respiratory tract infections. These results are consistent with and extend the findings of a preliminary epidemiological analysis of this association conducted in the US.^7^ That study found that among men aged 45 to 85 years (108 956 men from the MarketScan database and 252 708 men from the Optum database) who were hospitalized with pneumonia, the propensity score–matched odds ratio for in-hospital ventilation or death among those receiving vs not receiving α1-blockers was 0.91 (95% CI, 0.87-0.96).^7^ This finding is consistent with the RRs in the current study of 0.85 (95% CI, 0.83-0.88) for 30-day mortality and 0.92 (95% CI, 0.86-0.99) for mechanical ventilation associated with receipt vs nonreceipt of α1-blockers among patients with influenza or pneumonia.

Of interest, the US analysis also included 13 125 men from the Market Scan database and 6534 men from the Optum database who had a diagnosis code for acute respiratory distress, which is a potential precursor of acute respiratory distress syndrome. In this group, the association between α1-blocker receipt and protective benefits was clearer, with an adjusted odds ratio for in-hospital ventilation or death of 0.67 (95% CI, 0.46-0.96).^7^

In studies of mouse models, it has recently been reported that macrophages secrete and respond to catecholamines through adrenergic receptors when exposed to inflammatory stimuli such as bacteria. Catecholamines orchestrate cytokine production and severity of inflammation injury,^6^ and catecholamine synthesis inhibition reduces cytokine responses. When mice injected with bacterial lipopolysaccharide were pretreated with pharmacologic catecholamine blockade through metyrosine therapy, they were protected from the fatal complications of cytokine release syndrome.^6^

Emerging data from human studies suggest that a subset of patients with COVID-19 develops cytokine storm syndrome that is associated with increased production of proinflammatory cytokines (including interleukin 6, interleukin 2R, interleukin 8, tumor necrosis factor α, and granulocyte colony-stimulating factor)^8,18,19^ similar to the excessive cytokine production by lung-infiltrating pneumocytes and monocytes or macrophages observed in patients with severe acute respiratory coronavirus and Middle East respiratory syndrome coronavirus infections.^20^ Alveolar inflammation culminates in acute respiratory distress syndrome, which necessitates mechanical ventilation and is a main factor associated with COVID-19 mortality. Preventing hyperinflammation in an early phase seems important to avoid this progression,^4,5^ and the catecholamine pathway is a potential target for preventing hyperinflammation in patients with COVID-19. Randomized clinical trials will be needed to further test the hypothesis raised by animal experiments and epidemiological studies indicating that α1-blockers may be associated with decreases in the risks of cytokine storm syndrome and death among patients with COVID-19.

### Limitations

This study has several limitations. One limitation was the study’s reliance on diagnostic coding of influenza and pneumonia. Although some patients with these infections may not receive a diagnosis, we believe that restriction to physician-coded influenza and pneumonia discharge diagnoses ensured inclusion of only those patients with clinically relevant infections. The positive predictive value of pneumonia diagnoses in the Danish patient registry is 90%.^21,22^ Overall 30-day mortality after pneumonia diagnosis in this study’s population-based cohort was similar to that reported in other parts of the world.^23^ Any selection bias in this study should be minimal because follow-up was almost complete, and different associations between included and nonincluded patients would not be expected. Given the chronic receipt of the drugs included in the study, any misclassification from sporadic use that was not captured before hospitalization should be minor and not associated with the outcome of interest.

This study lacked data on continuous in-hospital use of α1-blockers, and possible drug discontinuation during acute illness may have produced an underestimation of any association. Deaths are accurately recorded in the Danish population registry and updated daily.^12^ Intensive care unit admissions and treatments are also accurately recorded, as the Danish patient registry is used for financial reimbursement to hospitals and for mandatory reporting to quality-of-care databases.^14^ Because conditions treated with α1-blockers(eg, BPH and hypertension) and severe influenza and pneumonia infections may both lead to acute hospitalization, conditioning the analyses on hospitalized patients may, in theory, introduce collider bias. However, in cases of influenza and pneumonia in which death occurs shortly after diagnosis, the infection is likely to be a main factor associated with hospitalization and death rather than an inconsequential variable. Moreover, this study found robust estimates among patients with a primary diagnosis of influenza or pneumonia and among patients with severe infections who were admitted to the ICU. Potential confounding by drug treatment indication was handled by using an active comparator in 1 analysis, using propensity score weighting that included a large number of potential confounders, and restricting analyses to subgroups according to treatment indication. Nevertheless, it is possible that unmeasured confounding factors impacted the study’s risk estimates. Healthy-user bias is an unlikely explanation for the findings, given that the comorbidities and lifestyle factors captured did not indicate healthier lifestyles among patients receiving α1-blockers. Moreover, patients who formerly received α1-blockers did not experience improved outcomes. Although the study included more than 500 000 patients, the precision of risk estimates was limited in some subgroups. Because more than 95% of patients receiving α1-blockers were men and more than 90% of people in Denmark are White, it remains uncertain whether these results will also apply to women and non-White individuals.

## Conclusions

In this study, patients receiving α1-blockers who were hospitalized with influenza or pneumonia had lower mortality after confounding factors were controlled for compared with those not receiving α1-blockers and those receiving 5α-reductase inhibitors. Thus, these data support the hypothesis that α1-blockers may have a clinically relevant association with outcomes among patients with acute respiratory tract infections. These findings will need to be reproduced among patients with confirmed COVID-19 infection. Randomized clinical trials may enable more definitive conclusions to be reached regarding the association between α1-blockers and ICU admission and mortality among patients with COVID-19 and other respiratory tract infections. Such clinical trials may evaluate any benefits associated with initiating treatment with α1-blockers early in the course of severe respiratory infection rather than the more chronic treatment examined in this study, and clinical trials may also consider possible adverse effects associated with α-1 blocker therapy.

## Notes

Supplement. **eTable 1.** Codes Used in the Study **eTable 2.** Characteristics of All Patients With Influenza and Pneumonia, Overall and by Main Outcomes **eTable 3.** Unadjusted Outcomes in Current Users of α1-Blockers Compared With Nonusers, Stratified by a Diagnosis of Any Influenza or Pneumonia, Only Influenza, or Pneumonia With Bacterial or Unspecified Pathogen **eTable 4.** Restriction to Male Patients: Risk of Different Outcomes in Male Current Users of α1-Blockers Compared With Male Nonusers, Adjusted by Propensity Score Weighting **eTable 5.** Characteristics of Current Male Users of α1-Blockers and 5α-Reductase Blockers Hospitalized With Influenza or Pneumonia, Overall and After Propensity Score Weighting **eTable 6.** Outcomes in Current Male Users of α1-Blockers Compared With Male Users of 5α-Reductase Blockers **eTable 7.** Outcomes in Current Users of Doxazosin, Alfuzosin, and Tamsulosin, Compared With Propensity Score–Weighted Nonusers of α1-Blockers **eFigure 1.** Study Design **eFigure 2.** Propensity Score Distributions Before and After Weighting: Current Users of α1-Blockers (Exposed) Compared With Nonusers (Unexposed)
