# Supplementary material for: Association of α1-Blocker Receipt With 30-Day Mortality and Risk of Intensive Care Unit Admission Among Adults Hospitalized With Influenza or Pneumonia in Denmark
Source: JAMA Netw Open. 2021 Feb 10;4(2):e2037053. doi: 10.1001/jamanetworkopen.2020.37053 (PMC7876591; doi:10.1001/jamanetworkopen.2020.37053)
Supplement: Supplement. — eTable 1. Codes Used in the Study eTable 2. Characteristics of All Patients With Influenza and Pneumonia, Overall and by Main Outcomes eTable 3. Unadjusted Outcomes in Current Users of α1-Blockers Compared With Nonusers, Stratified by a Diagnosis of Any Influenza or Pneumonia, Only Influenza, or Pneumonia With Bacterial or Unspecified Pathogen eTable 4. Restriction to Male Patients: Risk of Different Outcomes in Male Current Users of α1-Blockers Compared With Male Nonusers, Adjusted by Propensity Score Weighting eTable 5. Characteristics of Current Male Users of α1-Blockers and 5α-Reductase Blockers Hospitalized With Influenza or Pneumonia, Overall and After Propensity Score Weighting eTable 6. Outcomes in Current Male Users of α1-Blockers Compared With Male Users of 5α-Reductase Blockers eTable 7. Outcomes in Current Users of Doxazosin, Alfuzosin, and Tamsulosin, Compared With Propensity Score–Weighted Nonusers of α1-Blockers eFigure 1. Study Design eFigure 2. Propensity Score Distributions Before and After Weighting: Current Users of α1-Blockers (Exposed) Compared With Nonusers (Unexposed) [file jamanetwopen-e2037053-s001.pdf]

Supplementary Online Content

Thomsen RW, Christiansen CF, Heide-Jørgensen U, et al. Association of α1-blocker receipt with 30-day mortality and risk of intensive care unit admission among adults hospitalized with influenza or pneumonia in Denmark. *JAMA Netw Open*. 2021;4(2):e2037053. doi:10.1001/jamanetworkopen.2020.37053

eTable 1. Codes Used in the Study

eTable 2. Characteristics of All Patients With Influenza and Pneumonia, Overall and by Main Outcomes

eTable 3. Unadjusted Outcomes in Current Users of α1-Blockers Compared With Nonusers, Stratified by a Diagnosis of Any Influenza or Pneumonia, Only Influenza, or Pneumonia With Bacterial or Unspecified Pathogen

eTable 4. Restriction to Male Patients: Risk of Different Outcomes in Male Current Users of α1-Blockers Compared With Male Nonusers, Adjusted by Propensity Score Weighting

eTable 5. Characteristics of Current Male Users of α1-Blockers and 5α-Reductase Blockers Hospitalized With Influenza or Pneumonia, Overall and After Propensity Score Weighting

eTable 6. Outcomes in Current Male Users of α1-Blockers Compared With Male Users of 5α-Reductase Blockers

eTable 7. Outcomes in Current Users of Doxazosin, Alfuzosin, and Tamsulosin, Compared With Propensity Score–Weighted Nonusers of α1-Blockers

eFigure 1. Study Design

eFigure 2. Propensity Score Distributions Before and After Weighting: Current Users of α1-Blockers (Exposed) Compared With Nonusers (Unexposed)

This supplementary material has been provided by the authors to give readers additional information about their work.

© 2021 Thomsen RW et al. *JAMA Netw Open*.

eTable 1. Codes Used in the Study

Variable	Source/format	Look back	Inclusion codes	Exclusion codes
**Study population (ICD-10 codes)**				
Any influenza or pneumonia	DNPR/ICD-10		J09-J18, A481, B012, A709	
Influenza	DNPR/ICD-10		J09, J10	
Pneumonia with bacterial or unspecified pathogen	DNPR/ICD-10		J13, J14, J157, J159, J180, J181, J182, J189, A481	
**Main exposures (ATC codes)***				
Alpha-1 blockers	NPD/ATC	90d	G04CA, C02CA	
Alpha-1 blockers, urological	NPD/ATC	90d	G04CA	
alfuzosin			G04CA01	
tamsulosin			G04CA02	
terazosin			G04CA03	
silodosin			G04CA04	
alfuzosin and finasteride			G04CA51	
tamsulosin and dutasteride			G04CA52	
tamsulosin and solifenacin			G04CA53	
tamsulosin and tadalafil			G04CA54	
Alpha-1 blockers, antihypertensive	NPD/ATC	90d	C02CA	
prazosin			C02CA01	
indoramin			C02CA02	
trimazosin			C02CA03	
doxazosin			C02CA04	
urapidil			C02CA06	
**Outcomes**				
ICU admission	DNPR/Procedure		NABE, NABB	
Mechanical ventilation	DNPR/Procedure		BGDA0	
Non-invasive ventilation	DNPR/Procedure		BGDA1	
Treatment with inotropes/vasopressors	DNPR/Procedure		BFHC92A, BFHC92B, BFHC92C, BFHC92D, BFHC92E, BFHC93A, BFHC93B, BFHC93C, BFHC95	
Dialysis-treated acute kidney injury (“acute dialysis”)	DNPR/Procedure		BJFD0	
Death	CPR/status			
**Covariates**				
Age	CPR			
Sex	CPR			
**Coexisting conditions**				
Hospital-diagnosed hypertension	DNPR/ICD-10	10y	I10, I15	
Diagnosed stable angina pectoris	DNPR/ICD-10	10y	I20, I251, I259	I21, I22, I23, I200
Myocardial infarction	DNPR/ICD-10	10y	I21, I22, I23	

© 2021 Thomsen RW et al. *JAMA Netw Open*.

Heart failure	DNPR/ICD-10	10y	I50	
Stroke	DNPR/ICD-10	10y	I60, I61, I63, I64	
Atrial fibrillation/flutter	DNPR/ICD-10	10y	I48	
Heart valve disease	DNPR/ICD-10	10y	I05, I06, I07, I08, I09.8, I34-I37, I39, I51.1A, Q22, Q23	
Venous thromboembolism	DNPR/ICD-10	10y	I26, I801, I802, I803	
Diabetes	DNPR/ICD-10	10y	E10, E11, E12, E13, E14, O24, G63.2, H360, N083	O24.4
	NPD/ATC	10y	A10A, A10B	
Chronic pulmonary disease	DNPR/ICD-10	10y	J40, J41-J44, J45−J47, J60−J67, J68.4, J70.1, J70.3, J84.1, J92.0, J96.1, J98.2, J98.3	
Renal disease	DNPR/ICD-10	10y	I12, I13, N00−N05, N07, N11, N14, N18−N19, Q61, N08, E102, E112, E142	
End-stage renal disease (kidney transplant or dialysis)	DNPR/Procedure	10y	BJFD2	
	DNPR/Surgery	10y	KKAS	
	DNPR/ICD-10	10y	T861, Z940	
Liver disease	DNPR/ICD-10	10y	B18, B150, B160, B162, B190, I85, K70, K71, K72, K73, K74, K760, K76.6	
Dementia	DNPR/ICD-10	10y	DF00, DF01, DF02, DF03, DG30, DG310B, DG311, DG318, DG319	
	NPD/ATC	10y	N06D	
Cancer	DNPR/ICD-10	10y	C00−C96, D459, D46, D471, D473, D474, D475	C44
Metastatic cancer	DNPR/ICD-10	10y	C76−C80, CxxxM	
Peptic ulcer disease	DNPR/ICD-10	10y	K221, K25-K28	
Rheumatoid arthritis or connective tissue disease	DNPR/ICD-10	10y	M05-M06, M30–M36, M45	
**Comedications**				
Other benign prostatic hyperplasia (BPH) related drugs than alpha-1 blockers	NPD/ATC	90 d	G04CA51, G04CA52, G04CA53,G04CA54,G04CB , G04CX, G04BD08	
5-alpha reductase inhibitors	NPD/ATC	90 d	G04CB	
Other drugs used in BPH	NPD/ATC	90 d	G04CX	
Solifenacin for urgency			G04BD08	
Number of antihypertensive drugs	NPD/ATC	90d	0-5 (see below: ACE-I/ARB, CCB, Thiazides/diuretics, BB, other antihypertensive drugs)	
Angiotensin converting enzyme inhibitors (ACE-I)	NPD/ATC	90d	C09A, C09B	
Angiotensin II receptor blockers (ARB)	NPD/ATC	90d	C09C, C09D	

Calcium channel blockers (CCB)	NPD/ATC	90d	C07FB, C08CA, C09BB, C09DB, C09DX01	
Thiazides/diuretics	NPD/ATC	90d	C03AA01, C03AA03, C03AB01, C03AB03, C03AX01, C03EA, C07B, C09BA, C09DA, C09DX01, C09DX03, C09XA52, C09XA54	C07BA06 C09BA04
Beta-blockers (BB)	NPD/ATC	90d	C07	
Other antihypertensive drugs	NPD/ATC	90d	C02AC, C03BA11, C09XA C02AB01	C09XA54
Statins	NPD/ATC	90d	C10AA, C10B	
Aspirin	NPD/ATC	90d	B01AC06, N02BA01, N02BA51	
Loop diuretics	NPD/ATC	90d	C03C	
Antibiotics	NPD/ATC	10d	J01	
Antivirals	NPD/ATC	10d	J05	
Immunosuppressants	NPD/ATC	90d	L04	
Glucocorticoids	NPD/ATC	90d	H02AB	
Nonsteroidal anti-inflammatory drug (NSAID) use	NPD/ATC	90d	M01A	
Opioid use	NPD/ATC	90d	N02A, N07BC02	
Vitamin K antagonists	NPD/ATC	90d	B01AA	
Proton pump inhibitors	NPD/ATC	90d	A02BC	
Antidepressants	NPD/ATC	90d	N06A	
Hypnotics/sedatives	NPD/ATC	90d	N05C	
Antipsychotics	NPD/ATC	90d	N05A	
**Lifestyle and social factors**				
Markers of smoking (diagnoses or medications for tobacco smoking or chronic obstructive pulmonary disease)	DNPR/ICD-10	10y	J41-J44, DF17, DZ716, DZ720	
	NPD/ATC	10y	R03, N07BA	
Obesity diagnoses or medications	DNPR/ICD-10	10y	E66	
	NPD/ATC	10y	A08	
Alcoholism-related diagnoses or medication for alcohol deterrence	DNPR/ICD-10	10y	DF10, DE244, DG312, DG621, DG721, DI426, DK292, DK70, DK852, DK860, DQ860, DZ502, DZ714, DZ721	F100
	NPD/ATC	10y	V03AA, N07BB	
Marital status (widowed, divorced, married, umarried)	CPR			
Rural/urban place of residence	CPR			

Abbreviations: ATC, Anatomical Therapeutic Chemical Classification System; CPR, Danish Civil Reigistration System; DNPR, Danish National Patient Registry; ICD-10, *International Classification of Diseases.Tenth Revision*; NPD, National Prescription Database.

*Note on main exposure: Alpha-1-blockers (called Alpha-adrenoreceptor Antagonists in the ATC) are ATC classified both as antihypertensives and as BPH drugs. The ATC group “C02 Antihypertensives” includes category “C02CA Alpha-adrenoreceptor Antagonists”, which includes C02CA01 prazosin, C02CA02 indoramin, C02CA03 trimazosin, C02CA04 doxazosin, C02CA06 urapidil. The ATC group “G04C Drugs Used In BPH” includes category “G04CA Alpha-adrenoreceptor Antagonists”, which includes G04CA01 alfuzosin, G04CA02 tamsulosin, G04CA03 terazosin, G04CA04 silodosin, as well as the combination pills G04CA51 alfuzosin and finasteride, G04CA52 tamsulosin and dutasteride, G04CA53 tamsulosin and solifenacin, G04CA54 tamsulosin and tadalafil. In Denmark, for antihypertensive treatment, mainly doxazosin (“Carduran®” and others) from the “C02 Antihypertensives” group, as well as terazosin (“Hytrin” or “Sinalfa®”) from the “G04CA Drugs Used In BPH” group are used (https://pro.medicin.dk/Laegemiddelgrupper/grupper/97045). For BPH treatment, Doxazosin from the “C02 Antihypertensives” group, as well as virtually all drugs from the “G04CA Drugs Used In BPH” group are used.

© 2021 Thomsen RW et al. *JAMA Netw Open*.

eTable 2. Characteristics of All Patients With Influenza and Pneumonia, Overall and by Main Outcomes

Characteristic	*Death within 30 days*	*ICU admission*	*Overall study population*
Number of patients	77,192 (100.0)	41,276 (100.0)	528,467 (100.0)
Age, median (Q1 - Q3)	81.8 (73.2;88.0)	71.2 (61.9;79.0)	75.0 (64.4;83.6)
Male	41,954 (54.4)	23,995 (58.1)	273,005 (51.7)
**Comorbidity (within prior 10 years)**			
Hospital-diagnosed hypertension	27,104 (35.1)	13,098 (31.7)	169,907 (32.2)
Hospital diagnosis of benign prostatic hyperplasia	6,511 (8.4)	2,342 (5.7)	34,890 (6.6)
Previous myocardial infarction	6,697 (8.7)	3,229 (7.8)	40,034 (7.6)
Diagnosis of stable angina pectoris	10,615 (13.8)	4,953 (12.0)	68,928 (13.0)
Heart failure	13,851 (17.9)	5,746 (13.9)	71,515 (13.5)
Stroke	13,433 (17.4)	4,727 (11.5)	65,765 (12.4)
Atrial fibrillation/flutter	18,454 (23.9)	6,819 (16.5)	96,018 (18.2)
Heart valve disease	6,418 (8.3)	2,758 (6.7)	34,878 (6.6)
Venous thromboembolism	4,511 (5.8)	1,985 (4.8)	28,061 (5.3)
Diabetes	14,558 (18.9)	8,708 (21.1)	94,961 (18.0)
Chronic pulmonary disease	20,751 (26.9)	12,758 (30.9)	154,744 (29.3)
Renal disease	7,114 (9.2)	3,590 (8.7)	40,164 (7.6)
End-stage renal disease	971 (1.3)	751 (1.8)	8,391 (1.6)
Liver disease	2,488 (3.2)	2,315 (5.6)	14,511 (2.7)
Dementia	9,866 (12.8)	1,221 (3.0)	35,446 (6.7)
Cancer	21,526 (27.9)	7,067 (17.1)	106,640 (20.2)
Metastatic cancer	5,220 (6.8)	894 (2.2)	18,709 (3.5)
Peptic ulcer disease	6,139 (8.0)	3,184 (7.7)	32,475 (6.1)
Rheumatoid arhthritis or connective tissue disease	4,214 (5.5)	2,219 (5.4)	32,131 (6.1)
**Comedication**			
Alpha-1 blockers	3,451 (4.5)	1,596 (3.9)	21,772 (4.1)
Alpha-1 blockers, urological	2,968 (3.8)	1,284 (3.1)	18,405 (3.5)
Alfuzosin	1,021 (1.3)	442 (1.1)	6,200 (1.2)
Tamsulosin	1,881 (2.4)	808 (2.0)	11,700 (2.2)
Terazosin	72 (0.1)	36 (0.1)	510 (0.1)
Silodosin	0 (0.0)	0 (0.0)	0 (0.0)
Alfuzosin and finasteride	0 (0.0)	0 (0.0)	0 (0.0)
Tamsulosin and dutasteride	<5	<5	15 (0.0)
Tamsulosin and solifenacin	9 (0.0)	6 (0.0)	90 (0.0)
Tamsulosin and tadalafil	0 (0.0)	0 (0.0)	0 (0.0)
Alpha-1 blockers, antihypertensive	493 (0.6)	324 (0.8)	3,473 (0.7)
Prazosin	6 (0.0)	11 (0.0)	58 (0.0)
Indoramin	0 (0.0)	0 (0.0)	0 (0.0)
Trimazosin	0 (0.0)	0 (0.0)	0 (0.0)
Doxasosin	487 (0.6)	313 (0.8)	3,415 (0.6)
Urapidil	0 (0.0)	0 (0.0)	0 (0.0)
BPH-related drugs other than alpha-1 blockers	1,521 (2.0)	509 (1.2)	7,845 (1.5)
5-alpha reductase inhibitors	1,509 (2.0)	503 (1.2)	7,749 (1.5)

© 2021 Thomsen RW et al. *JAMA Netw Open.*

Characteristic	*Death within 30 days*	*ICU admission*	*Overall study population*
Total number of antihypertensives			
0	39,848 (51.6)	21,533 (52.2)	283,052 (53.6)
1	21,824 (28.3)	9,872 (23.9)	130,384 (24.7)
2	11,318 (14.7)	6,616 (16.0)	80,981 (15.3)
3-6	4,202 (5.4)	3,255 (7.9)	34,050 (6.4)
Angiotensin converting enzyme inhibitors	11,977 (15.5)	7,217 (17.5)	82,239 (15.6)
Angiotensin II receptor blockers	6,396 (8.3)	4,198 (10.2)	53,463 (10.1)
Calcium channel blockers	10,168 (13.2)	6,551 (15.9)	71,234 (13.5)
Thiazides	11,377 (14.7)	6,270 (15.2)	79,942 (15.1)
Beta-blockers	17,483 (22.6)	9,037 (21.9)	111,434 (21.1)
Other antihypertensives	417 (0.5)	334 (0.8)	2,944 (0.6)
Statins	14,012 (18.2)	9,293 (22.5)	113,579 (21.5)
Aspirin	20,873 (27.0)	9,308 (22.6)	120,524 (22.8)
Loop diuretics	26,900 (34.8)	10,730 (26.0)	128,180 (24.3)
Antibiotics	18,135 (23.5)	7,111 (17.2)	142,019 (26.9)
Antivirals	140 (0.2)	64 (0.2)	1,230 (0.2)
Immunosuppressants	712 (0.9)	544 (1.3)	6,430 (1.2)
Gluccorticoids	13,917 (18.0)	5,707 (13.8)	77,680 (14.7)
Nonsteroidal anti-inflammatory drugs	9,152 (11.9)	5,776 (14.0)	64,327 (12.2)
Opioids	28,632 (37.1)	10,648 (25.8)	142,665 (27.0)
Vitamin K antagonists	5,345 (6.9)	3,031 (7.3)	38,167 (7.2)
Proton pump inhibitors	25,948 (33.6)	10,763 (26.1)	145,017 (27.4)
Antidepressants	21,566 (27.9)	9,200 (22.3)	118,399 (22.4)
Hypnotics/sedatives	13,979 (18.1)	6,169 (14.9)	78,198 (14.8)
Antipsychotics	7,691 (10.0)	3,493 (8.5)	37,879 (7.2)
**Lifestyle and social factors**			
Markers of smoking	34,796 (45.1)	21,137 (51.2)	265,346 (50.2)
Obesity	3,636 (4.7)	3,739 (9.1)	39,825 (7.5)
Alcoholism	6,574 (8.5)	6,780 (16.4)	44,632 (8.4)
Marital status			
Widowed	31,023 (40.2)	9,393 (22.8)	154,558 (29.2)
Divorced	10,647 (13.8)	7,736 (18.7)	84,145 (15.9)
Married	28,437 (36.8)	18,169 (44.0)	234,192 (44.3)
Unmarried	7,085 (9.2)	5,978 (14.5)	55,572 (10.5)
Urban residence	26,628 (34.5)	12,734 (30.9)	187,439 (35.5)

© 2021 Thomsen RW et al. *JAMA Netw Open*.

eTable 3. Unadjusted Outcomes in Current Users of α1-Blockers Compared With Nonusers, Stratified by a Diagnosis of Any Influenza or Pneumonia, Only Influenza, or Pneumonia With Bacterial or Unspecified Pathogen

Population	*Event*	*Current alpha-1 blocker use events/at risk*	*Current alpha-1 blocker use Risk %*	*Alpha-1 blocker non-use events/at risk*	*Alpha-1 blocker non-use Risk %*	*Risk difference % (95% CI) vs alpha-1 blocker non-use*	*Risk ratio (95% CI) vs alpha-1 blocker non-use*
**Any influenza or pneumonia**	Death	3 451 / 21 772	15.9	72 035 / 497 576	14.5	1.4 (0.9 - 1.8)	1.09 (1.06 - 1.13)
	ICU admission	1 596 / 21 772	7.3	38 967 / 497 576	7.8	-0.5 (-0.9 - -0.1)	0.94 (0.89 - 0.98)
	ICU + MV	804 / 21 772	3.7	21 353 / 497 576	4.3	-0.6 (-0.9 - -0.3)	0.86 (0.80 - 0.92)
	ICU + NIV	666 / 21 772	3.1	14 961 / 497 576	3.0	0.1 (-0.2 - 0.3)	1.02 (0.94 - 1.10)
	ICU + inotropes	715 / 21 772	3.3	17 195 / 497 576	3.5	-0.2 (-0.4 - 0.1)	0.95 (0.89 - 1.02)
	D-AKI	214 / 21 085	1.0	4 141 / 490 260	0.8	0.2 (0.0 - 0.3)	1.20 (1.04 - 1.39)
**Influenza**	Death	24 / 327	7.3	415 / 7 309	5.7	1.7 (-1.3 - 4.6)	1.29 (0.86 - 1.95)
	ICU admission	30 / 327	9.2	663 / 7 309	9.1	0.1 (-3.1 - 3.3)	1.01 (0.71 - 1.44)
	ICU + MV	12 / 327	3.7	449 / 7 309	6.1	-2.5 (-4.5 - -0.5)	0.60 (0.34 - 1.05)
	ICU + NIV	18 / 327	5.5	318 / 7 309	4.4	1.2 (-1.5 - 3.8)	1.27 (0.77 - 2.08)
	ICU + inotropes	12 / 327	3.7	377 / 7 309	5.2	-1.5 (-3.5 - 0.5)	0.71 (0.41 - 1.22)
	D-AKI	[a]	[a]	[a]	[a]	-1.1 (-1.9 - -0.4)	0.23 (0.09 - 0.60)
**Bacterial pneumonia or unspecified pathogen**	Death	3 256 / 20 360	16.0	67 843 / 460 257	14.7	1.3 (0.7 - 1.8)	1.08 (1.05 - 1.12)
	ICU admission	1 483 / 20 360	7.3	36 280 / 460 257	7.9	-0.6 (-0.9 - -0.3)	0.92 (0.88 - 0.97)
	ICU + MV	742 / 20 360	3.6	19 691 / 460 257	4.3	-0.6 (-0.9 - -0.4)	0.85 (0.80 - 0.91)
	ICU + NIV	613 / 20 360	3.0	13 980 / 460 257	3.0	0.0 (-0.2 - 0.2)	0.99 (0.92 - 1.06)
	ICU + inotropes	663 / 20 360	3.3	15 902 / 460 257	3.5	-0.2 (-0.4 - 0.0)	0.94 (0.87 - 1.02)
	D-AKI	196 / 19 736	1.0	3 771 / 453 610	0.8	0.2 (0.0 - 0.3)	1.19 (1.04 - 1.38)

[a]To ensure anonymity, Danish legislation prohibits reporting of exact n= measures where low n results (e.g. n<5) are observed or can be inferred in other categories.

Abbreviations: ICU, Intensive care unit; MV, Mechanical ventilation; NIV, Non-invasive ventilation; D-AKI, Dialysis-treated acute kidney injury .

© 2021 Thomsen RW et al. *JAMA Netw Open.*

eTable 4. Restriction to Male Patients: Risk of Different Outcomes in Male Current Users of α1-Blockers Compared With Male Nonusers, Adjusted by Propensity Score Weighting

Population	*Event*	*Current alpha-1 blocker use events/at risk*	*Current alpha-1 blocker use Risk %*	*Alpha-1 blocker non-use events/at risk*	*Alpha-1 blocker non-use Risk %*	*Risk difference % (95% CI) vs alpha-1 blocker non-use*	*Risk ratio (95% CI) vs alpha-1 blocker non-use*
**Any influenza or pneumonia**	Death	3 354 / 20 984	16.0	4 001 / 21 266	18.8	-2.8 (-3.4 - -2.3)	0.85 (0.82 - 0.88)
	ICU admission	1 530 / 20 984	7.3	1 638 / 21 266	7.7	-0.4 (-0.8 - 0.0)	0.95 (0.90 - 1.00)
	ICU + MV	774 / 20 984	3.7	845 / 21 266	4.0	-0.3 (-0.5 - 0.0)	0.93 (0.86 - 1.00)
	ICU + NIV	633 / 20 984	3.0	646 / 21 266	3.0	0.0 (-0.3 - 0.2)	0.99 (0.91 - 1.08)
	ICU + inotropes	689 / 20 984	3.3	730 / 21 266	3.4	-0.1 (-0.4 - 0.1)	0.96 (0.89 - 1.03)
	D-AKI	202 / 20 405	1.0	184 / 20 663	0.9	0.1 (0.0 - 0.2)	1.11 (0.96 - 1.29)
**Influenza**	Death	22 / 320	6.9	25 / 328	7.6	-0.7 (-4.4 - 3.0)	0.91 (0.10 - 8.20)
	ICU admission	28 / 320	8.8	23 / 328	6.8	1.9 (-1.8 - 5.6)	1.28 (0.82 - 2.00)
	ICU + MV	11 / 320	3.4	14 / 328	4.1	-0.6 (-3.0 - 1.7)	0.84 (0.10 - 7.31)
	ICU + NIV	16 / 320	5.0	11 / 328	3.2	1.8 (-0.9 – 4.6)	1.58 (0.17 - 15.08)
	ICU + inotropes	12 / 320	3.8	14 / 328	4.3	-0.5 (-3.0 – 2.0)	0.88 (0.09 - 8.17)
	D-AKI	[a]	[a]	[a]	[a]	-0.3 (-1.1 - 0.4)	0.51 (0.19 - 1.42)
**Bacterial/unspecified pneumonia**	Death	3 163 / 19 617	16.1	3 771 / 19 869	19.0	-2.9 (-3.4 - -2.3)	0.85 (0.82 - 0.88)
	ICU admission	1 421 / 19 617	7.2	1 526 / 19 869	7.7	-0.4 (-0.8 - 0.0)	0.94 (0.89 - 1.00)
	ICU + MV	713 / 19 617	3.6	781 / 19 869	3.9	-0.3 (-0.6 - 0.0)	0.92 (0.86 – 1.00)
	ICU + NIV	582 / 19 617	3.0	605 / 19 869	3.0	-0.1 (-0.3 - 0.2)	0.97 (0.89 - 1.06)
	ICU + inotropes	637 / 19 617	3.2	677 / 19 869	3.4	-0.2 (-0.4 - 0.1)	0.95 (0.88 - 1.03)
	D-AKI	184 / 19 093	1.0	168 / 19 325	0.9	0.1 (0.0 - 0.2)	1.11 (0.95 - 1.29)

© 2021 Thomsen RW et al. *JAMA Netw Open*.

eTable 5. Characteristics of Current Male Users of α1-Blockers and 5α-Reductase Blockers Hospitalized With Influenza or Pneumonia, Overall and After Propensity Score Weighting

	Overall cohort			***Propensity score weighted cohort***		
	Current alpha-1 blocker use, N (%)	***Current 5-alpha reductase blocker use, N (%)***	***SD***	***Current alpha-1 blocker use, N (%)***	***Current 5-alpha reductase blocker use, N (%)***	***SD***
Number of patients (males)	18 280 (80.7)	4 382 (19.3)		18 280 (50.1)	18 228 (49.9)	
Age, median (Q1 - Q3)	79.4 (72.6;85.2)	83.5 (78.1;88.1)	0.68	79.4 (72.6;85.2)	79.4 (72.7;85.1)	0.00
**Comorbidity (within prior 10 years)**						
Hospital-diagnosed hypertension	7 730 (42.3)	1 805 (41.2)	0.03	7 730 (42.3)	7 256 (39.8)	0.07
Hospital diagnosis of benign prostatic hyperplasia	4 721 (25.8)	1 839 (42.0)	0.49	4 721 (25.8)	4 698 (25.8)	0.00
Previous myocardial infarction	1 832 (10.0)	513 (11.7)	0.08	1 832 (10.0)	1 680 (9.2)	0.04
Diagnosis of stable angina pectoris	3 601 (19.7)	893 (20.4)	0.02	3 601 (19.7)	3 701 (20.3)	0.02
Heart failure	3 280 (17.9)	972 (22.2)	0.15	3 280 (17.9)	3 359 (18.4)	0.02
Stroke	2 855 (15.6)	769 (17.5)	0.07	2 855 (15.6)	2 775 (15.2)	0.02
Atrial fibrillation/flutter	4 754 (26.0)	1 332 (30.4)	0.14	4 754 (26.0)	4 689 (25.7)	0.01
Heart valve disease	1 691 (9.3)	455 (10.4)	0.05	1 691 (9.3)	1 676 (9.2)	0.00
Venous thromboembolism	996 (5.4)	232 (5.3)	0.01	996 (5.4)	976 (5.4)	0.01
Diabetes	4 513 (24.7)	847 (19.3)	0.18	4 513 (24.7)	4 236 (23.2)	0.05
Chronic pulmonary disease	6 091 (33.3)	1 389 (31.7)	0.05	6 091 (33.3)	6 052 (33.2)	0.00
Renal disease	2 592 (14.2)	473 (10.8)	0.15	2 592 (14.2)	2 238 (12.3)	0.08
End-stage renal disease	544 (3.0)	41 (0.9)	0.21	544 (3.0)	388 (2.1)	0.08
Liver disease	347 (1.9)	52 (1.2)	0.08	347 (1.9)	331 (1.8)	0.01
Dementia	1 350 (7.4)	465 (10.6)	0.16	1 350 (7.4)	1 343 (7.4)	0.00
Cancer	4 572 (25.0)	1 017 (23.2)	0.06	4 572 (25.0)	4 771 (26.2)	0.04
Metastatic cancer	663 (3.6)	164 (3.7)	0.01	663 (3.6)	663 (3.6)	0.00
Peptic ulcer disease	1 301 (7.1)	318 (7.3)	0.01	1 301 (7.1)	1 305 (7.2)	0.00
Rheumatoid arthritis or connective tissue disease	1 060 (5.8)	171 (3.9)	0.12	1 060 (5.8)	1 072 (5.9)	0.00
**Comedications**						
BPH-related drugs other than alpha-1 blockers	94 (0.5)	*	.	94 (0.5)	*	.
Total number of antihypertensives						
0	7 447 (40.7)	1 762 (40.2)	0.02	7 447 (40.7)	7 658 (42.0)	0.04
1	5 454 (29.8)	1 376 (31.4)	0.05	5 454 (29.8)	5 464 (30.0)	0.00
2	3 647 (20.0)	880 (20.1)	0.00	3 647 (20.0)	3 528 (19.4)	0.02
3-6	1 732 (9.5)	364 (8.3)	0.06	1 732 (9.5)	1 577 (8.7)	0.04
Angiotensin converting enzyme inhibitors	3 743 (20.5)	943 (21.5)	0.04	3 743 (20.5)	3 588 (19.7)	0.03
Angiotensin II receptor blockers	2 364 (12.9)	529 (12.1)	0.04	2 364 (12.9)	2 284 (12.5)	0.02
Calcium channel blockers	3 473 (19.0)	722 (16.5)	0.09	3 473 (19.0)	3 277 (18.0)	0.04
Thiazides	3 256 (17.8)	754 (17.2)	0.02	3 256 (17.8)	3 161 (17.3)	0.02
Beta-blockers	5 295 (29.0)	1 314 (30.0)	0.03	5 295 (29.0)	5 123 (28.1)	0.03
Other antihypertensives	226 (1.2)	38 (0.9)	0.05	226 (1.2)	174 (1.0)	0.04
Statins	5 621 (30.7)	1 377 (31.4)	0.02	5 621 (30.7)	5 601 (30.7)	0.00
Aspirin	5 966 (32.6)	1 490 (34.0)	0.04	5 966 (32.6)	5 887 (32.3)	0.01
Loop diuretics	6 185 (33.8)	1 553 (35.4)	0.05	6 185 (33.8)	6 149 (33.7)	0.00
Antibiotics	4 682 (25.6)	1 097 (25.0)	0.02	4 682 (25.6)	4 602 (25.2)	0.01
Antivirals	31 (0.2)	6 (0.1)	0.01	31 (0.2)	21 (0.1)	0.02
Immunosuppressants	225 (1.2)	41 (0.9)	0.04	225 (1.2)	231 (1.3)	0.00
Gluccorticoids	3 204 (17.5)	657 (15.0)	0.10	3 204 (17.5)	3 051 (16.7)	0.03
Nonsteroidal anti-inflammatory drugs	2 298 (12.6)	468 (10.7)	0.08	2 298 (12.6)	2 353 (12.9)	0.01
Opioids	5 207 (28.5)	1 146 (26.2)	0.07	5 207 (28.5)	5 381 (29.5)	0.03
Vitamin K antagonists	2 159 (11.8)	584 (13.3)	0.06	2 159 (11.8)	2 124 (11.7)	0.01
Proton pump inhibitors	6 128 (33.5)	1 448 (33.0)	0.01	6 128 (33.5)	5 917 (32.5)	0.03
Antidepressants	4 425 (24.2)	1 063 (24.3)	0.00	4 425 (24.2)	4 392 (24.1)	0.00
Hypnotics/sedatives	2 980 (16.3)	666 (15.2)	0.04	2 980 (16.3)	3 151 (17.3)	0.04
Antipsychotics	1 243 (6.8)	297 (6.8)	0.00	1 243 (6.8)	1 294 (7.1)	0.02
**Lifestyle and social factors**						
Markers of smoking	9 739 (53.3)	2 184 (49.8)	0.10	9 739 (53.3)	9 651 (52.9)	0.01
Obesity	1 180 (6.5)	183 (4.2)	0.14	1 180 (6.5)	1 162 (6.4)	0.00
Alcoholism	1 291 (7.1)	173 (3.9)	0.19	1 291 (7.1)	1 357 (7.4)	0.02
Marital status						
Widowed	4 248 (23.2)	1 328 (30.3)	0.23	4 248 (23.2)	4 220 (23.2)	0.00
Divorced	2 039 (11.2)	363 (8.3)	0.14	2 039 (11.2)	2 040 (11.2)	0.00
Married	10 650 (58.3)	2 461 (56.2)	0.06	10 650 (58.3)	10 571 (58.0)	0.01

© 2021 Thomsen RW et al. *JAMA Netw Open.*

	Overall cohort			***Propensity score weighted cohort***		
	Current alpha-1 blocker use, N (%)	***Current 5-alpha reductase blocker use, N (%)***	***SD***	***Current alpha-1 blocker use, N (%)***	***Current 5-alpha reductase blocker use, N (%)***	***SD***
Unmarried	1 343 (7.3)	230 (5.2)	0.12	1 343 (7.3)	1 396 (7.7)	0.02
Urban residence	5 504 (30.1)	1 424 (32.5)	0.07	5 504 (30.1)	5 527 (30.3)	0.01

© 2021 Thomsen RW et al. *JAMA Netw Open*.

eTable 6. Outcomes in Current Male Users of α1-Blockers Compared With Male Users of 5α-Reductase Blockers

Population	***Event***	***Current alpha-1 blocker use events/at risk***	***Current alpha-1 blocker use Risk %***	***5-alpha reductase blocker use events/at risk***	***5-alpha reductase blocker use Risk %***	***Risk difference % (95% CI) vs 5-alpha reductase blocker use***	***Risk ratio (95% CI) vs 5-alpha reductase blocker use***
Any influenza or pneumonia	death	2 921 / 18 280	16.0	3 276 / 18 227	18.0	-2.0 (-3.4 - -0.6)	0.89 (0.82 - 0.96)
	ICU	1 357 / 18 280	7.4	1 416 / 18 227	7.8	-0.3 (-1.4 - 0.7)	0.96 (0.83 - 1.10)

© 2021 Thomsen RW et al. *JAMA Netw Open*.

eTable 7. Outcomes in Current Users of Doxazosin, Alfuzosin, and Tamsulosin, Compared With Propensity Score–Weighted Nonusers of α1-Blockers

A: Current doxazosin vs α1-blocker nonuse

Population	***Event***	***Current doxazosin use events/at risk***	***Current doxazosin use Risk %***	***Alpha-1 blocker non-use events/at risk***	***Alpha-1 blocker non-use Risk %***	***Risk difference % (95% CI) vs alpha-1 blocker non-use***	***Risk ratio (95% CI) vs alpha-1 blocker non-use***
Any influenza or pneumonia	Death	487 / 3 415	14.3	533 / 3 441	15.5	-1.2 (-2.4 - -0.1)	0.92 (0.85 - 1.00)
	ICU	313 / 3 415	9.2	314 / 3 441	9.1	0.0 (-1.0 - 1.1)	1.00 (0.90 - 1.12)

B: Current alfuzosin vs α1-blocker nonuse

Population	***Event***	***Current alfuzosin use events/at risk***	***Current alfuzosin use Risk %***	***Alpha-1 blocker non-use events/at risk***	***Alpha-1 blocker non-use Risk %***	***Risk difference % (95% CI) vs alpha-1 blocker non-use***	***Risk ratio (95% CI) vs alpha-1 blocker non-use***
Any influenza or pneumonia	Death	1 021 / 6 200	16.5	1 180 / 6 225	19.0	-2.5 (-3.4 - -1.6)	0.87 (0.82 - 0.92)
	ICU	442 / 6 200	7.1	473 / 6 225	7.6	-0.5 (-1.2 - 0.3)	0.94 (0.85 - 1.04)

C: Current tamsulosin vs α1-blocker nonuse

Population	***Event***	***Current tamsulosin use events/at risk***	***Current tamsulosin use Risk %***	***Alpha-1 blocker non-use events/at risk***	***Alpha-1 blocker non-use Risk %***	***Risk difference % (95% CI) vs alpha-1 blocker non-use***	***Risk ratio (95% CI) vs alpha-1 blocker non-use***
Any influenza or pneumonia	Death	1 881 / 11 700	16.1	2 259 / 11 803	19.1	-3.1 (-3.8 - -2.3)	0.84 (0.80 - 0.88)
	ICU	808 / 11 700	6.9	886 / 11 803	7.5	-0.6 (-1.1 - -0.1)	0.92 (0.86 - 0.99)

© 2021 Thomsen RW et al. *JAMA Netw Open*.

eFigure 1. Study Design

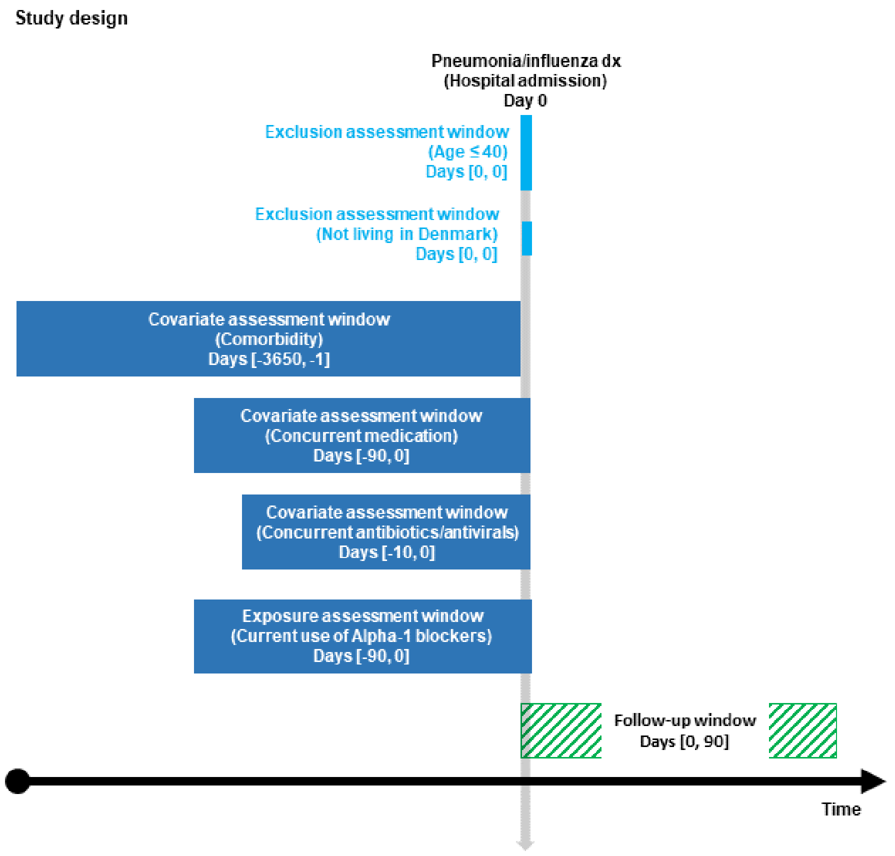

© 2021 Thomsen RW et al. *JAMA Netw Open*.

eFigure 2. Propensity Score Distributions Before and After Weighting: Current Users of α1-Blockers (Exposed) Compared With Nonusers (Unexposed)

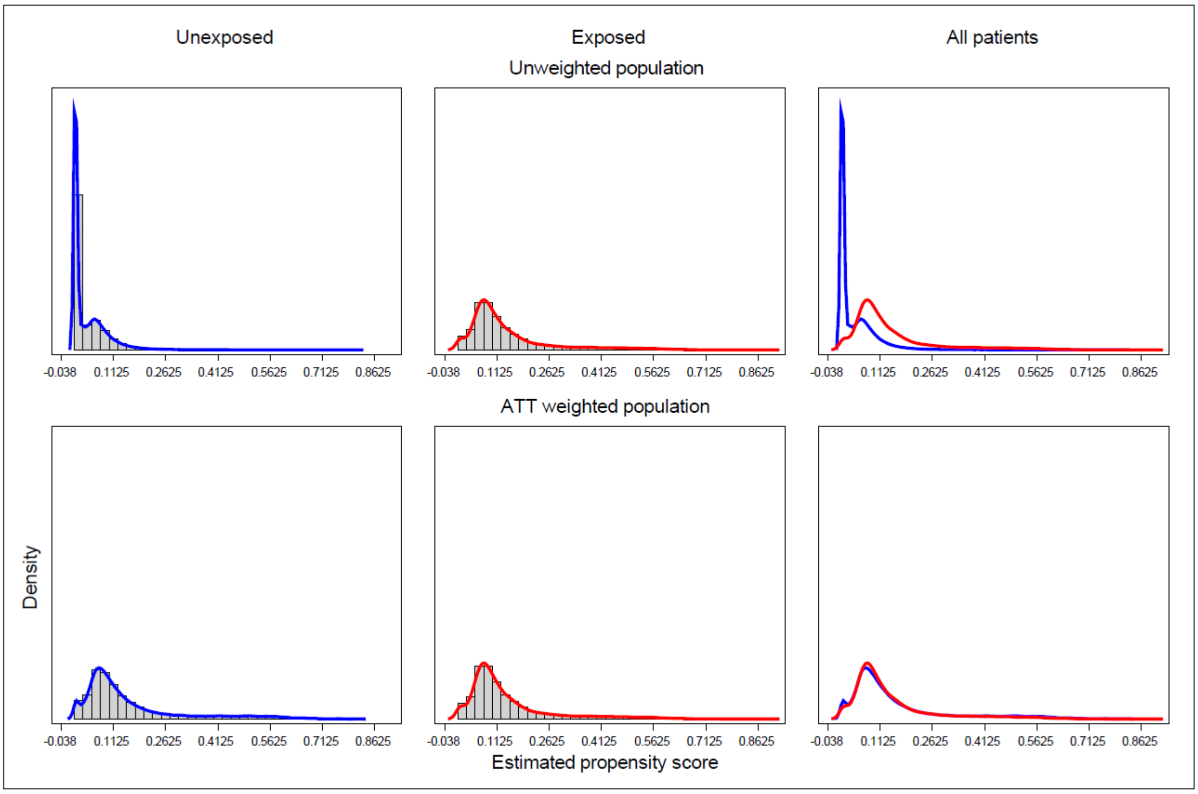

© 2021 Thomsen RW et al. *JAMA Netw Open*.
